# Is Mitochondrial tRNA^phe^ Variant m.593T>C a Synergistically Pathogenic Mutation in Chinese LHON Families with m.11778G>A?

**DOI:** 10.1371/journal.pone.0026511

**Published:** 2011-10-19

**Authors:** A-Mei Zhang, Hans-Jürgen Bandelt, Xiaoyun Jia, Wen Zhang, Shiqiang Li, Dandan Yu, Dong Wang, Xin-Ying Zhuang, Qingjiong Zhang, Yong-Gang Yao

**Affiliations:** 1 Key Laboratory of Animal Models and Human Disease Mechanisms of Chinese Academy of Sciences and Yunnan Province, Kunming Institute of Zoology, Kunming, Yunnan, China; 2 State Key Laboratory of Ophthalmology, Zhongshan Ophthalmic Center, Sun Yat-sen University, Guangzhou, Guangdong, China; 3 Department of Mathematics, University of Hamburg, Hamburg, Germany; 4 Graduate School of the Chinese Academy of Sciences, Beijing, China; Hospital Universitario 12 de Octubre, Spain

## Abstract

Mitochondrial transfer RNA (mt-tRNA) mutations have been reported to be associated with a variety of diseases. In a previous paper that studied the mtDNA background effect on clinical expression of Leber's hereditary optic neuropathy (LHON) in 182 Chinese families with m.11778G>A, we found a strikingly high frequency (7/182) of m.593T>C in the mitochondrially encoded tRNA phenylalanine (*MT-TF*) gene in unrelated LHON patients. To determine the potential role of m.593T>C in LHON, we compared the frequency of this variant in 479 LHON patients with m.11778G>A, 843 patients with clinical features of LHON but without the three known primary mutations, and 2374 Han Chinese from the general populations. The frequency of m.593T>C was higher in LHON patients (14/479) than in suspected LHON subjects (12/843) or in general controls (49/2374), but the difference was not statistically significant. The overall penetrance of LHON in families with both m.11778G>A and m.593T>C (44.6%) was also substantially higher than that of families with only m.11778G>A (32.9%) (*P* = 0.083). Secondary structure prediction of the *MT-TF* gene with the wild type or m.593T>C showed that this nucleotide change decreases the free energy. Electrophoretic mobility of the *MT-TF* genes with the wild type or m.593T>C transcribed *in vitro* further confirmed the change of secondary structure in the presence of this variant. Although our results could suggest a modest synergistic effect of variant m.593T>C on the LHON causing mutation m.11778G>A, the lack of statistical significance probably due to the relatively small sample size analyzed, makes necessary more studies to confirm this effect.

## Introduction

Leber's hereditary optic neuropathy (LHON; MIM 535000) is characterized by acute or sub-acute visual failure in young adults and is the first mitochondrial disorder described [1], [2]. It later turned out that in over 95% of LHON cases the disease was caused by the presence of one of three primary mutations that were located in the *MT-ND4* gene (m.11778G>A), the *MT-ND1* gene (m.3460G>A), and the *MT-ND6* gene (m.14484T>C), respectively. Incomplete penetrance and gender bias are two features of the clinical expression of LHON, but the exact underlying mechanisms for the onset of these two features have not been well resolved. Nuclear genes, mtDNA background/haplogroups, and environmental factors have been shown or suggested to affect the penetrance of LHON [1], [2].

Human mitochondrial transfer RNAs (mt-tRNAs) are essential for translation of the thirteen mtDNA encoded protein subunits. Twenty-two mt-tRNAs are transcribed from mtDNA, with one corresponding to one amino acid (excluding leucine and serine), and cannot be imported into mitochondria from the cytoplasm in human [3]. Mutations in mt-tRNAs, either in a sporadic status or maternally inherited, constitute the most common mtDNA alterations that are associated with human disorders [4]. Polymorphisms in mt-tRNAs are also common in general populations. Hitherto, more than 100 mt-tRNA mutations have been reported to be associated with mitochondrial disorders [5]. Among them, m.3243A>G in the *MT-TL1* [mitochondrially encoded tRNA leucine 1 (UUA/G)] gene is one of the most common mt-tRNA mutations that cause a variety of human diseases, such as diabetes, mitochondrial myopathy, MELAS (mitochondrial myopathy, encephalopathy, lactic acidosis, and stroke-like episodes) [6], [7]. The pathogenic mutations in mt-tRNA can alter the secondary structure or change one highly conserved base to another base, abolish the tertiary structure and lead to dysfunction [5], [8], [9]. Mutations in mt-tRNAs affect biogenesis and function of mt-tRNAs via a large variety of mechanisms, including transcription, maturation, posttranscriptional modification, structure, stability, aminoacylation, capability of binding to elongation factor EF-Tu, and codon reading [10].

In a recent study we showed that mtDNA haplogroups M7b1′2 and M8a affected the clinical expression of LHON in Chinese families with m.11778G>A, with an increased risk for M7b1′2 and a decreased risk for M8a [11]. This pattern was different from that of western European LHON patients, in which other haplogroups appear to contribute to the increased risk of visual failure in families with m.11778G>A (haplogroup J2), m.14484T>C (haplogroup J1), or m.3460G>A (haplogroup K), whereas haplogroup H had a protective effect for families with m.11778G>A [12]. Intriguingly, we found that variant m.593T>C in tRNA^phe^ (*MT-TF*) had a relatively high occurrence in Chinese LHON patients (7/182) [11]. The distribution frequency of m.593T>C in Chinese LHON patients with m.11778G>A was by an order of magnitude higher than in East Asian populations (4/1262, http://www.phylotree.org/; Table S1). In order to determine whether an association of m.593T>C with LHON could be predicted, we have systematically screened m.593T>C in 479 LHON patients with m.11778G>A (including 175 mtDNAs from the earlier study [11]), 843 patients suspected with LHON, and 2374 Han Chinese from the general populations without any visual disorder. Complete mtDNA sequencing of probands with both m.593T>C and m.11778G>A and *in vitro* transcribed assay were further performed to understand the potential interaction between the variant and LHON. Our results suggest that m.593T>C in LHON families may have a potentially synergistic effect with m.11778G>A.

## Materials and Methods

### Ethics statement

Written informed consents conforming to the tenets of the Declaration of Helsinki were obtained from each participant prior to this study. The institutional review boards of the Zhongshan Ophthalmic Center and the Kunming Institute of Zoology approved this study.

### Patients

We recently launched a comprehensive survey for mtDNA mutations in Chinese patients with LHON or suspected LHON and eventually provided the largest patient sample collection in China to date. Part of this patient cohort was characterized in a series of previous publications [11], [13]–[18]. Among this cohort, 479 LHON patients harbored m.11778G>A (including 175 mtDNAs from the earlier study [11]), 843 patients had clinical features of LHON but without the three known primary mutations and were termed suspected LHON patients. All these patients were collected at the Pediatric and Genetic Clinic of the Eye Hospital, Zhongshan Ophthalmic Center or other local ophthalmic centers. A total of 2374 Han Chinese who had no ophthalmologic disease were collected from Yunan and Hunan Province and were screened for the presence of m.593T>C. Because the matrilineal structure of patients with suspected LHON was similar to that of the general population (authors' unpublished data), we therefore took the 843 suspected LHON patients collected from different provinces in China as another control population for comparison. We followed our previous study [14] to define as sporadic any proband from a family with only one patient (according to the accessible pedigree information) or with unclear family history. In particular, this definition for sporadic case also includes the family with several asymptomatic carriers of m.11778G>A but with only one affected member (viz. the proband).

### mtDNA sequence analysis

Total genomic DNA was isolated from blood using a standard phenol/chloroform method. The mtDNA control region sequences and complete mtDNA genomes were amplified and sequenced using a modified method as described in our previous studies [11], [19]. Sequences were handled by the DNASTAR program (DNASTAR Inc., Madison, WI, USA). Sequence variation was scored relative to the revised Cambridge Reference Sequence (rCRS) [20]. We classified the LHON mtDNAs using PhyloTree Build 11 (http://www.phylotree.org; 7 Feb 2011) [21] and MitoTool (http://www.mitotool.org) [22]. mtDNA variants in each complete mtDNA were scored as novel or reported according to an exhaustive database search following previous guidelines [23]. Five reported mtDNA sequences (GenBank accession nos. GQ301863 [24], AF347007 [25], AY255137 [26], EF153821 [27], and AP008571 [28]) from East Asian populations with m.593T>C were considered for comparison. Sequence variation of each mtDNA relative to the rCRS was presented in an mtDNA tree, following the same procedure as in our previous studies [11], [29]. Evolutionary conservation analysis for m.593T>C mutation was performed using the same approach as in our previous study [19]. The potential relationship among these mtDNAs harboring m.593T>C that were identified from the LHON patients, suspected LHON patients and Han Chinese from the general populations was presented in a network, following the approach described in Bandelt et al. [30].

### Site-directed mutagenesis and in vitro transcription of the *MT-TF* gene

The wild type human mt-tRNA^Phe^ plasmid and two mutant plasmids (G7A hmt-tRNA^Phe^ [bearing m.583G>A] and G34A hmt-tRNA^Phe^ [bearing m.611G>A]) were kind gifts from Dr. Michael Ibba's lab [9]. Site-directed mutagenesis was performed to obtain mt-tRNA^Phe^ mutant plasmid bearing m.593T>C. The mutant primers were T593C-F (GTTTATGTAGCTTACCCCCTCAAAGCAATACACT) and T593C-R (AGTGTATTGCTTTGAGGGGGTAAGCTACATAAAC). PCR reaction was performed in a volume of 50 µL reaction mixture containing 5 µL 10×Cloned Pfu DNA polymerase reaction buffer (containing 2 mM Mg^2+^), 2.5 units of PfuTurb hotstart DNA polymerase (Stratagene), 400 µM of each dNTP, 0.1 µM of each primer, and 50 ng wild type mt-tRNA^Phe^ plasmid DNA. PCR amplification cycles were composed of one denaturation cycle at 94°C for 5 min, 30 cycles of 94°C for 30 s, 65°C for 40 s and 72°C for 4 min, one final extension cycle at 72°C for 10 min.

The PCR product was transformed into DH5α competent cells (Tiangen Bio CO. LTD., Beijing, China) and the plasmids were amplified. We purified the plasmids by using TIANprep Mini Plasmid Kit (Tiangen Bio CO. LTD., Beijing, China). The wild type mt-tRNA^Phe^ and mutant mt-tRNA^Phe^ plasmids were transcribed *in vitro* by using mMESSAGE mMACHINE Kit (Ambion, Inc) following the manufacture's instruction.

### Analysis of *MT-TF* secondary structure

The secondary structures of the *MT-TF* gene with or without m.593T>C were analyzed by the MFOLD program (http://mobyle.pasteur.fr/cgi-bin/portal.py) [31] to predict the potential change caused by the nucleotide alteration. Native and denaturing gel electrophoresis were used to detect the structure change of the *MT-TF* gene caused by m.593T>C. The two reported pathogenic mutations (m.583G>A [32] and m.611G>A [33]) in the *MT-TF* gene and the wild-type *MT-TF* gene were used as positive controls and negative control [9], respectively. The transcribed *MT-TF* RNA was separated by PAGE following the same condition described by Ling et al. [9]. In brief, native gel was comprised of 1×TBE (89 mM Tris-boric/2 mM Na_2_EDTA, pH 8.3) and 12% acrylamide-bis, and was run at 50 V at 4°C for 11 hours. Denaturing gel was comprised of 7 M urea and 12% acrylamide-bis, and was run at 200 V at room temperature for 1 hour.

### Statistical analysis

Two tailed Fisher's exact test was used to evaluate the difference of m.593T>C frequency in LHON samples with m.11778G>A and suspected LHON samples or normal controls. The penetrance rates of LHON in pedigrees with LHON family history and m.11778G>A in the presence or absence of m.593T>C were also quantified. A *P* value less than 0.05 was regarded as statistically significant.

## Results

### Clinical features of LHON families with both m.11778G>A and m.593T>C

A total of 14 LHON patients from six difference provinces who had both m.11778G>A and m.593T>C were distilled from the entire patients cohort. Among them, seven patients with a family history (Le51, Le251, Le394, Le549, Le554, Le953 and Le1120) were reported in our previous study [11], seven sporadic patients were newly included in this study (Table 1). The occurrence of m.593T>C in LHON patients with m.11778G>A (2.92%; 14/479) was two-fold higher than that of suspected LHON samples (1.42%; 12/843), but statistical analysis only showed a *P* value marginally close to 0.05 (*P* = 0.066). The higher frequency of m.593T>C in LHON patients with m.11778G>A is mainly due to the 175 reported matrilines from pedigrees with a family history of LHON [11] (we counted one proband per family). In fact, when these mtDNAs were excluded we found that the frequency of m.593T>C in the remaining 304 LHON samples (2.3%; 7/304) was close to that of Han Chinese from the general populations (2.06%; 49/2374). Because the majority of these 304 mtDNAs were from patients with sporadic LHON, it is most likely that m.593T>C was only enriched in patients from pedigrees with a family history of LHON. Note that we grouped these patients from a family with only one patient (according to the accessible pedigree information) or with unclear family history as sporadic in this study. This working definition may cause a potential bias for the above comparison.

**Table 1:** Detailed information of LHON probands with m.593T>C.

Sample	Haplogroup	Location	Family history	No. of affected family members	Total number of family members	Penetrance(%)
Le53	M7b1′2′4	Guangdong	No	-	-	-
Le682	G1c	Guangdong	No	-	-	-
Le840	D4g2	Jiangxi	No	-	-	-
Le878	G1c	Jiangxi	No	-	-	-
Le1192	B5a1c1	Guangdong	No	-	-	-
Le1407	B5a1c1	Hunan	No	-	-	-
Le1561	G1c	Guangdong	No	-	-	-
Le51^a^	C7a2	Guangdong	Yes	5	8	62.5
Le251^a^	Z	Henan	Yes	5	5	100.0
Le394^a^	D4	Hebei	Yes	4	14	28.6
Le549^a^	M10a2	Anhui	Yes	4	10	40.0
Le554^a^	G1c	Henan	Yes	2	9	22.2
Le953^a^	G1c	Hebei	Yes	3	5	60.0
Le1120^a^	D4g2	Jiangxi	Yes	2	5	40.0

a These seven LHON families were reported in our previous study [11].

The penetrance of LHON in the seven pedigrees that presented a family history and harbored both m.11778G>A and m.593T>C was 44.6% (25/56) (Table 1), much higher than that of families with only m.11778G>A (32.9%, 594/1803) [11]. However, this difference was not significant either (*P* = 0.083), partially because of the relatively small sample size of families with both m.11778G>A and m.593T>C.

### mtDNA sequence variation and evolutionary analysis

**Figure 1:**
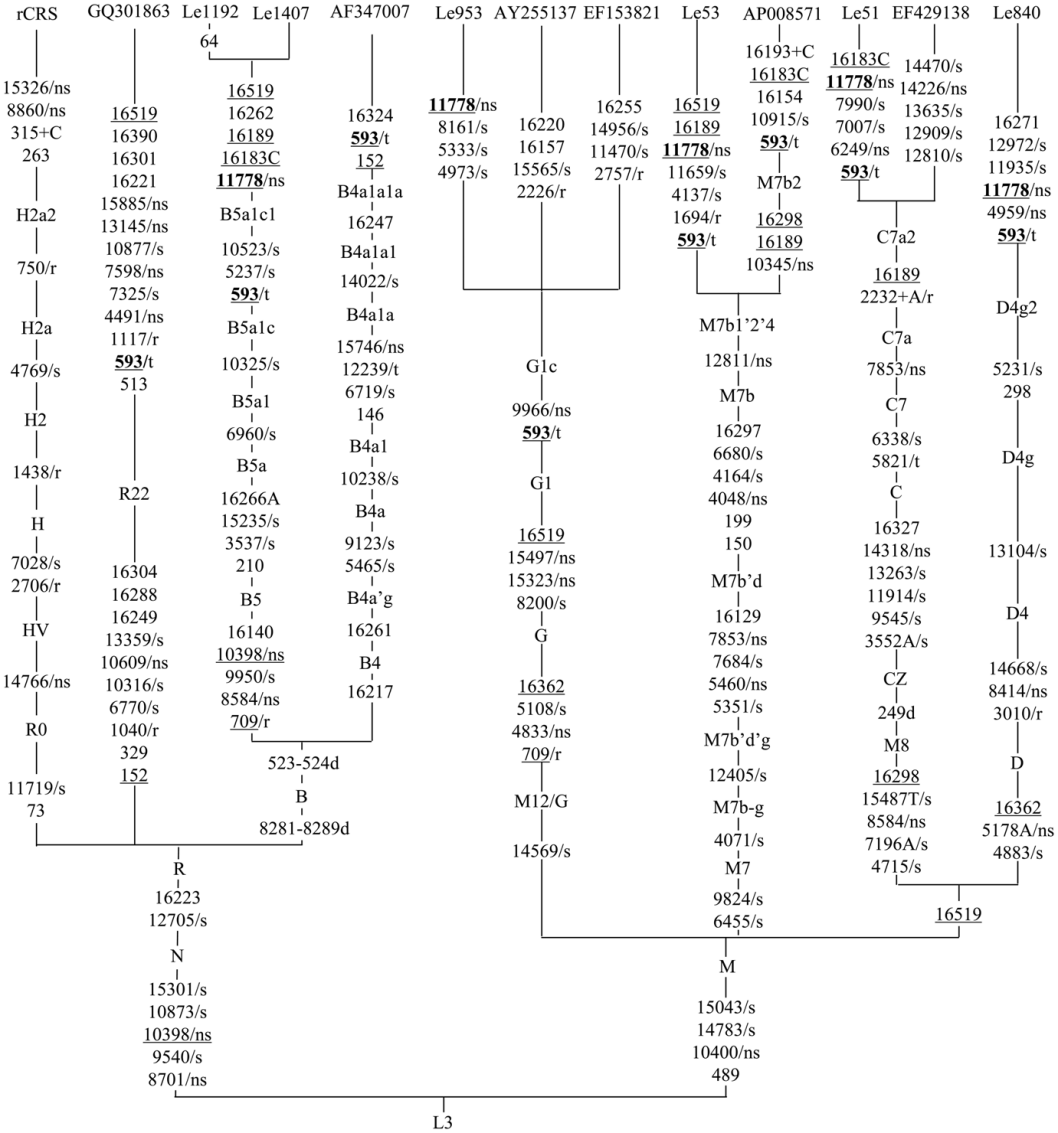
Classification tree of 12 complete mtDNA sequences, plus the revised Cambridge reference sequence (rCRS) [**20**]. Six Chinese LHON mtDNAs (including Le953 [FJ198218] [11]) had both m.593T>C and m.11778G>A. Five reported mtDNAs (GQ301863 [24], AF347007 [25], AY255137 [26], EF153821 [27], and AP008571 [28]) harbored m.593T>C. One mtDNA (EF429138 [43]) without either variant was used to define the novel haplogroup C7a2. The length polymorphisms of the C-tracts in region 303–309 were disregarded. The order of mutations on each uninterrupted branch segment is arbitrary. Recurrent mutations are underlined. The synonymous and non-synonymous coding-region variants in the mtDNA sequences are denoted by “/s” and “/ns”, respectively. Variants in the ribosomal RNA genes and tRNA genes are denoted by “/r” and “/t”, respectively.

**Figure 2:**
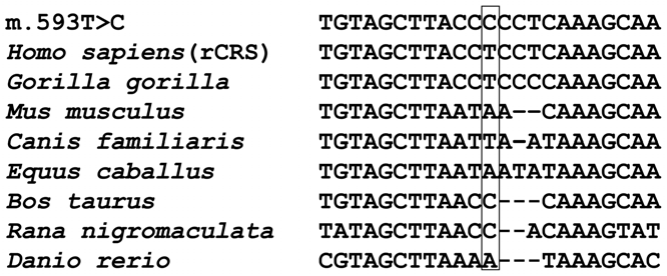
Evolutionary conservation analysis for mitochondrial tRNA^Phe^ variant m.593T>C. The mt-tRNA sequence with m.593T>C is compared to the revised Cambridge reference sequence (rCRS; *Homo sapiens*, GenBank accession number J01415) and those derived from seven different vertebrate species: gorilla (*Gorilla gorilla* NC_001645), mouse (*Mus musculus* AY466499), dog (*Canis familiaris* DQ480502), cattle (*Bos taurus* AY526085), horse (*Equus caballus* EF597513), zebrafish (*Danio rerio* NC_002333), and frog (*Rana nigromaculata* AB043889).

We sequenced the entire mtDNA genomes of five LHON patients (Le51, Le53, Le840, Le1192, and Le1407; GenBank accession numbers JF896797–JF896801) and the mtDNA control region sequences of three probands (Le682, Le878 and Le1561) with both m.11778G>A and m.593T>C (Table 1). Sequence variation (in either the complete mtDNA genome or in the control region) of the remaining six LHON patients was reported in our previous study [11]. According to mtDNA sequence variation (Table S2), patients Le682, Le878 and Le1561 belonged to haplogroup G1c. Analysis of the five complete mtDNA sequences indicated that these lineages belonged to haplogroups C7a2 (newly defined here based on the motif 16189-2232+A, Le51), M7b1′2′4 (Le53), D4g2 (Le840), and B5a1c1 (newly defined here based on the motif 593-5237-10325-10523, Le1192 and Le1407), respectively (Fig. 1). Besides m.11778G>A, m.593T>C and the haplogroup-specific variants in each sample, there were several private variants in each lineage (Table 2). Le51 harbored five private variants (m.6249G>A and m.7007C>T in the *MT-CO1* gene, m.7990C>T in the *MT-CO2* gene, and m.16183A>C and m.16519T>C in the control region). Among these, only variant m.6249G>A (p.A116T) causes an amino acid change. Le53 owned five private variants (m.1694T>C in the *MT-RNR2* gene, the synonymous changes m.4137C>T in the *MT-ND1* gene and m.11659C>T in the *MT-ND4* gene, and m.16189T>C and m.16519T>C in the control region). Le840 had five private variants that were located in the *MT-ND2* (m.4959G>A, p.A164T), *MT-ND4* (m.11935T>C), *MT-ND5* (m.12972A>G) genes, and the control region (m.16271T>C and m.16519T>C). Le1192 also had several private variants including three synonymous changes in the coding region (m.5237G>A in the *MT-ND2* gene, m.10325G>A in the *MT-ND3* gene, m.10523A>G in *MT-ND4L* gene) and six variants in the control region (m.64C>T, m.523-524d, m.16183A>C, m.16189T>C, m.16262C>T and m.16519T>C). There was no novel mtDNA variant [23] in these lineages. None of the three private non-synonymous variants and one mt-tRNA variant that were identified in the above patients was conserved, and all of them were reported in general populations, suggesting that these variants were most likely polymorphisms (Table 2). Similarly, the thymine at the 17th position (i.e., m.593T) of the *MT-TF* gene, which is located in the dihydrouracil loop (D loop) of tRNA^Phe^, was not conserved in eight vertebrate species (Fig. 2).

**Table 2:** Private non-synonymous and mt-tRNA variants in Chinese LHON mtDNAs with m.11778G>A and m.593T>C.

Family^a^	Haplogroup	Nucleotide variant (Amino acid change)	Gene	Conservation^c^	Reported(Population context)^b^	Reported(Disease context)^b^	Haplogroup specific variant^d^
Le51	C7a2	m.6249G>A (p.A116T)	*MT-COI*	No	Yes	Yes	L0d2c, P7
Le53	M7b1′2′4	m. 1694T>C	*MT-RNR2*	No	Yes	No	L4b2a2
Le840	D4g2	m. 4959G>A (p.A164T)	*MT-ND2*	No	Yes	Yes	D4h3a4, D4e1a2a, T1a2

a The complete mtDNA complete genomes of Le1192 and Le1407 contained no private non-synonymous and mt-tRNA variants and were not included.

b The search was performed on April 18, 2011 following the same strategy described earlier [23] (e.g. both ‘G6249A mtDNA’ and ‘m.6249G>A mtDNA’ were queried).

c The conservation analysis was performed by comparing *Homo sapiens* mtDNA (GenBank accession No. J01415) to 43 different vertebrate species by using the MitoTool (http://www.mitotool.org) [22].

d The column “Haplogroup specific variant” refers to the presence of the corresponding variants in the world mtDNA phylogeny displayed at http://www.phylotree.org/tree/main.htm (mtDNA tree Build 11; 7 Feb 2011) [21]. In round brackets we indicate the haplogroup status as it defined in that tree.

**Figure 3:**
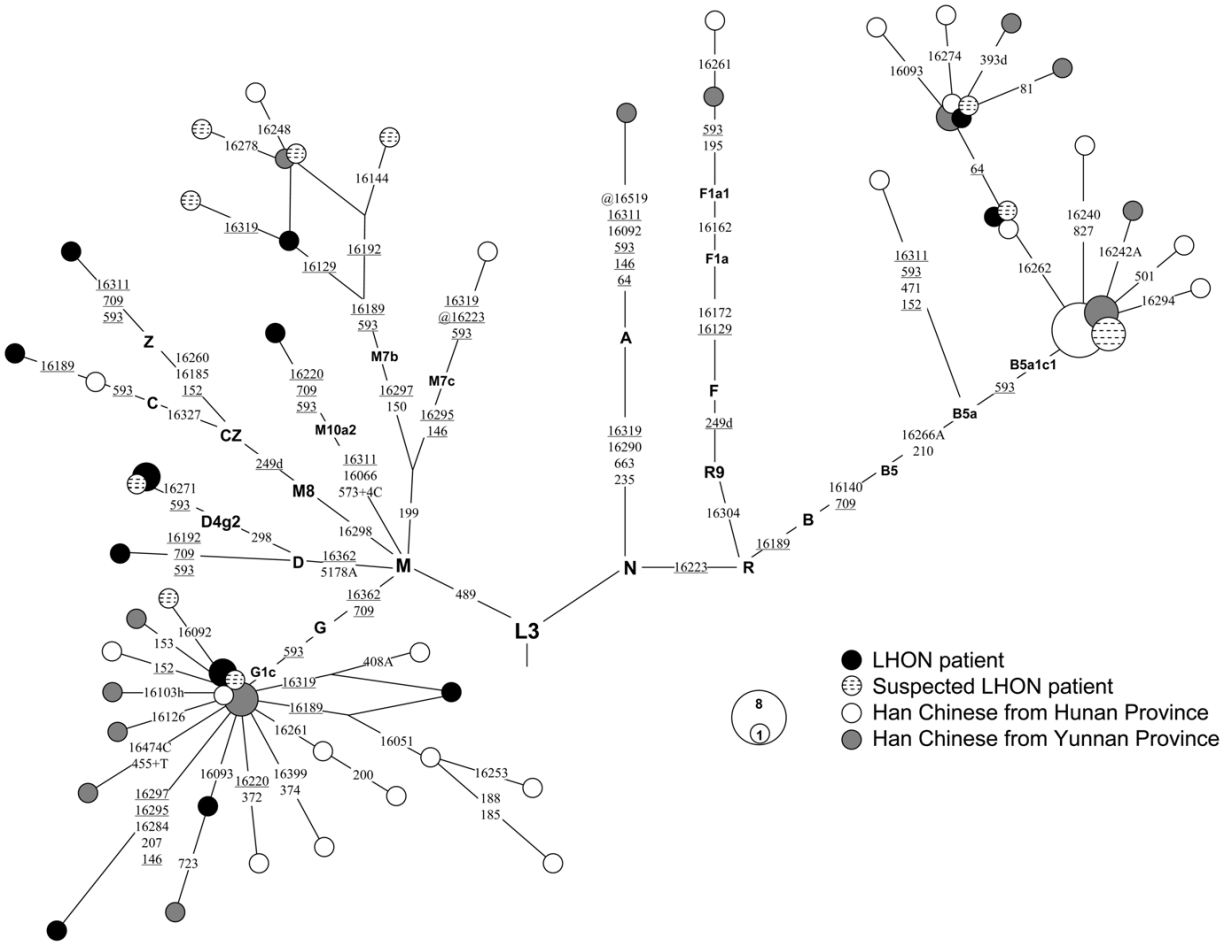
Network of 75 mtDNAs with m.593T>C that were identified in 479 LHON patients, 843 suspected LHON patients and 2374 Han Chinese from the general populations. Each circle represents an mtDNA haplotype, with its area being proportional to the frequency of the haplotype. The order of mutations on each uninterrupted branch section is arbitrary. Recurrent mutations are underlined. The length polymorphisms of the C-tracts in regions 16183–16192 and 303–309 and of AC repeats in region 515–524 in the mtDNA control region were disregarded. All individuals contain variants 16519-73-263-315+C-750 relative to the revised Cambridge reference sequence (rCRS) [20]. The current classification within M7b will need some revision in the future in regard to the positions 16189 and 16192, so that the exact number of 593 mutational events is left undetermined for the time being.

We constructed a network for all the 75 mtDNAs with m.593T>C that were identified from the LHON patients (n = 14), suspected LHON patients (n = 12) and Han Chinese from the general populations (n = 49). It is evident that m.593T>C occurred multiple times in different haplogroups, and it defined haplogroups G1c and B5a1c1 (Fig. 3 and Table S2).

### *MT-TF* gene secondary structure analysis

**Figure 4:**
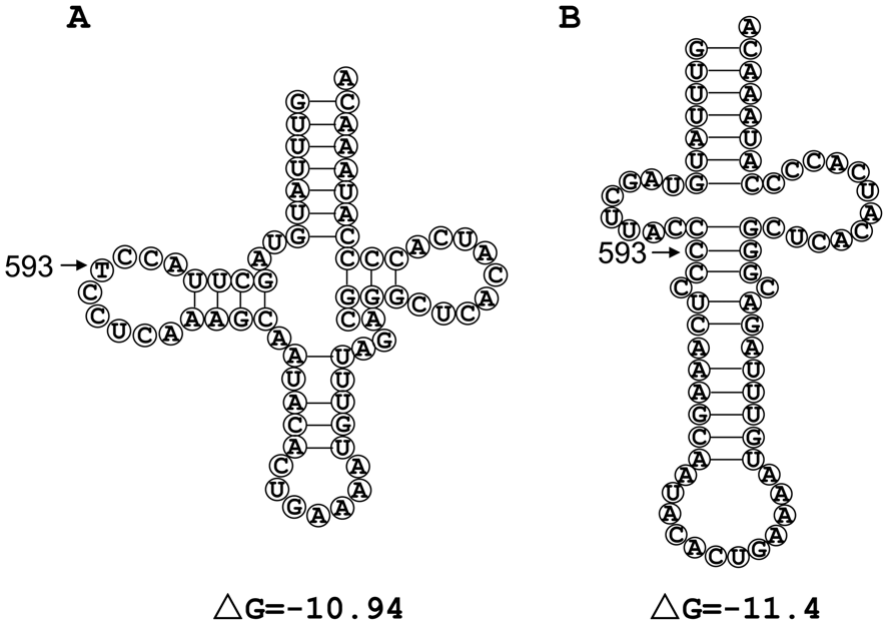
Predicted secondary structure of the wild type human *MT-TF* gene (A) and the mutant harboring m.593T>C (B). Position 593 was marked by an arrow. ΔG means the free energy.

The free energy is a criterion for judging the stability of RNA structure *in vivo*; most of the RNA secondary structure predictions are based on the free energy minimization method [32], [34]. The RNA secondary structure can be predicted more accurately by thermodynamics determined from the primary sequence without information of tertiary contacts or protein interaction; the lower the free energy the more stable the structure is, but this is not an absolute fact because of biological complexity [35]. Alteration of the *MT-TF* gene secondary structure in the presence of m.593T>C is shown in Figure 4. The predicted structure of the wild type *MT-TF* gene has a free energy (ΔG) value of −10.94 (Fig. 4A). There are two predicted structures of the *MT-TF* gene bearing variant m.593T>C. The first type is similar to that of the wild type gene, whereas the second one has a lower free energy value of −11.4 and a reduced size of the dihydrouracil loop (D loop) (Fig. 4B). We speculate that the shift of two predicted structures of the *MT-TF* gene *in vivo* in the presence of m.593T>C might slightly impair its function, despite the fact that the second predicted structure had a lower free energy (which means higher stability).

**Figure 5:**
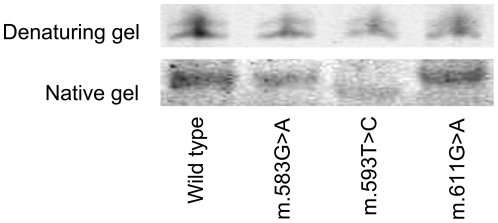
Native and denaturing gels showing the migration of RNAs of the wild type *MT-TF* gene and mutants. Electrophoretic mobility is from top to bottom. The mutant tRNA^Phe^ plasmids with m.583G>A and m.611G>A are gifts from Prof. Michael Ibba's lab and were named as G7A hmt-tRNA^Phe^ and G34A hmt-tRNA^Phe^ in their study [9], respectively.

The electrophoretic mobility of the secondary structure of the wild type and mutant *MT-TF* genes transcribed *in vitro* showed that variant m.593T>C affected the migration of mutant tRNA^Phe^ compared to the wild type on the native gel. However, this structure change disappeared when we separated the transcribed RNAs on a denaturing gel (Fig. 5). The observed pattern was in good agreement with the predicted change of the secondary structure of the *MT-TF* gene in the presence of m.593T>C.

## Discussion

Mitochondrial tRNAs played an important role in mtDNA translation because the nuclear tRNA could not be transported from cytoplasm into mitochondria in human [3], [36]. The mutations occurred in mt-tRNAs can change the secondary structure and alter the tertiary structure, further affect the translation of mtDNA encoded genes. Many pathogenic mutations in mt-tRNA genes have been reported to be associated with human diseases, despite that defining the pathogenicity of mt-tRNA mutations are not easy [4], [5], [8], [10], [37]. In this study, we aimed to elucidate the role of variant m.593T>C in the *MT-TF* gene in LHON patients. By screening m.593T>C in 479 LHON cases with m.11778G>A (including 175 cases reported in our earlier study [11]), 843 suspected LHON samples without the three known primary mutations, as well as 2374 general controls, we observed a higher frequency of m.593T>C in LHON patients with m.11778G>A (2.92%; 14/479) than in suspected LHON patients (1.42%; 12/843) or Han Chinese from the general populations (2.06%; 49/2374), despite that the difference was not statistically significant. However, when we focused on 182 matrilines from 182 Chinese families (including 7 families analyzed in other studies; Ref. [11] and see references therein; we counted only one affected member per family) with m.11778G>A and a family history of LHON [11], the frequency of m.593T>C (3.85%; 7/182) was substantially higher than those of suspected LHON patients and Han Chinese from the general populations. This suggests that m.593T>C was only enriched in pedigrees with m.11778G>A and a family history of LHON, but not in sporadic LHON patients with m.11778G>A.

In the presence of mtDNA subhaplogroups defined by m.593T>C (among other variants), hidden population substructure may influence the result. Because variant m.593T>C belongs to one of the characteristic motifs of both haplogroups G1c and B5a1c1 (Fig. 1), we therefore excluded those samples with m.593T>C belonging to these two haplogroups, to minimize the potentially regional effect on the distribution of m.593T>C in our samples. After removing these samples, we found a substantially higher frequency m.593T>C in LHON patients with m.11778G>A (1.48%; 7/472) than in suspected LHON patients (0.60%; 5/836) or in Han Chinese from the general populations (0.30%; 7/2332). Another potential effect could in principle be caused by haplogroup M7b1′2 which was shown to increase the risk of visual loss in the presence of m 11778G>A [11]. However, there was only a single LHON sample from this haplogroup in our study bearing m.593T>C, so that this haplogroup could not have biased our present results.

In concordance with the increased frequency of m.593T>C in LHON patients with m.11778G>A, we found that the seven LHON pedigrees with m.11778G>A, m.593T>C and a family history of the disease had a higher penetrance (44.6%) than that of pedigrees with only m.11778G>A (32.9%), albeit this difference was not statistically significant. This is another piece of evidence that the relatively high occurrence of m.593T>C among patients with a family history of LHON would be unlikely to be a mere chance event. More pedigrees with both m.11778G>A and m.593T>C are essential to further validate this pattern.

In a recent study by Kaewsutthi et al. [38], haplogroup B5a1 was suggested to increase risk of visual loss in LHON patients with m.11778G>A from Thailand. However, only three of the reported 10 B5a1 LHON lineages shared 10325 with our newly defined B5a1c. The relatively lower frequency of B5a in LHON patients (0.4%) than those of suspected LHON patients (0.6%) and Han Chinese from general populations (1.0%) indicates that this haplogroup is unlikely to affect LHON in Chinese.

Recent evaluation of mtDNA mutation rate showed that position 593 had a modest mutation rate [39] and is not evolutionarily conserved in vertebrate species (Fig. 2). Analysis of the complete mtDNA sequences in 6 patients (including the reported Le953 [11]) revealed no previously unreported private variants (Table 2 and Fig. 1). It is therefore conceivable that the relatively higher penentrance of LHON in those families with m.593T>C and m.11778G>A was not caused by the private variants in each lineage but rather by the synergistic effect of m.593T>C in the presence of m.11778G>A.

Despite that evolutionary analysis of m.593T>C showed that this position is not conserved, secondary structure prediction and *in vitro* experiment of the *MT-TF* gene demonstrated that the mutant allele changes the secondary structure *in vitro* (Figs. 4 and 5). It is possible that this structure alteration affects the efficiency of cognition between the phenylalanyl-tRNA synthetase (PheRS) and tRNA^Phe^ and protein translation. Some pathogenic mutations in the *MT-TF* gene, such as m.582T>C and m.583G>A, can change the secondary structure of the *MT-TF* gene and decrease the aminoacylation activity after transcribed *in vitro* [9]. Two classes of aminoacyl tRNA synthetase (I and II) with different editing sites have been identified [40]. The PheRS belongs to class II and its editing site is located in the B3/B4 domain [41]. mt-tRNA^Phe^ with m.593T>C may not be cognized by the PheRS because this variant changed the secondary tertiary structure of the *MT-TF* gene. Moreover, editing activity of the PheRS decreased during the evolution of mitochondrial PheRS, and the decreased cognitional activity between the PheRS and tRNA^Phe^ may affect the translational quality control [42]. The mutant tRNA^Phe^ caused by m.593T>C may decrease the cognitional activity to the PheRS and further enhance the clinical expression of LHON in the presence of m.11778G>A. Further functional assays, e.g. a test for the translation efficiency in cybrid cells with m.11778G>A mtDNA carrying or not this variant, are essential for validating our speculation and for understanding the potential mechanism under the presumed synergistic effect of m.593T>C with m.11778G>A.

In summary, we found a higher distribution frequency of m.593T>C in LHON patients with m.11778G>A than in the control populations, though the difference was not statistically significant. Presence of m.593T>C increased the penetrance of LHON in families with m.11778G>A, albeit the difference also did not reach a statistically significant level (probably due to the limited number of samples). Despite that the position 593 was not evolutionarily conserved and had a modest mutation rate in human mtDNA, it altered the secondary structure of the *MT-TF* gene as demonstrated by an *in vitro* transcribed assay. All these lines of evidence suggest that m.593T>C may enact a modest (if any) synergistic effect with m.11778G>A in LHON. More studies are needed to validate our current findings and to unveil the effect of the *MT-TF* gene variant on the pathogenicity of mutation m.11778G>A.

## Supporting Information

Table S1 Presence of variant m.593T>C in 1262 East Asian mtDNAs from the PhyloTree database.
(DOC)

Table S2 mtDNA sequence variation of 75 individuals with m.593T>C.
(PDF)
