# Supplementary material for: Is Mitochondrial tRNAphe Variant m.593T>C a Synergistically Pathogenic Mutation in Chinese LHON Families with m.11778G>A?
Source: PLoS One. 2011 Oct 19;6(10):e26511. doi: 10.1371/journal.pone.0026511 (PMC3198432; doi:10.1371/journal.pone.0026511)
Supplement: Table S1 — Presence of variant m.593T>C in 1262 East Asian mtDNAs from the PhyloTree database. (DOC) [file pone.0026511.s001.doc]

Table S1. Presence of variant m.593T>C in 1262 East Asian mtDNAs from the PhyloTree database

| Region | No. of complete mtDNAs | Occurrence of m.593T>C | GenBank accession number ^a^ | Reference |
| --- | --- | --- | --- | --- |
| Mainland China | 48 | 1 | AY255137 | Kong *et al.*^1^ |
| Mainland China | 20 | 0 |  | Kong *et al.*^2^ |
| Mainland China | 1 | 0 |  | Bandelt *et al.*^3^ |
| Mainland China | 10 | 0 |  | Wang *et al.*^4^ |
| Taiwan, China | 8 | 0 |  | Trejaut *et al.^5^* |
| Japan | 672 | 1 | AP008571 | Tanaka *et al.*^6^; Kong et al. ^7^ |
| Japan | 57 | 0 |  | Kazuno *et al.*^8^ |
| Japan | 112 | 0 |  | Bilal *et al.*^9^ |
| Japan | 14 | 0 |  | Nohira *et al.*^10^ |
| Japan | 90 | 0 |  | Ueno *et al.*^11^ |
| Korea | 4 | 1 | EF153821 | Derenko *et al.*^12^ |
| Tibet, China | 25 | 0 |  | Zhao *et al.*^13^ |
| Vietnam | 28 | 1 | GQ301863 | Peng *et al.*^14^ |
| Zhejiang, China | 1 | 0 |  | Bi *et al.*^15^ |
| China | 51 | 0 |  | Kong *et al.*^16^ |
| China | 59 | 0 |  | Peng *et al.*^17^ |
| Tibet, China | 31 | 0 |  | Qin *et al.^18^* |
| China & Japan | 13 | 0 |  | Hartmann *et al.* ^19^ |
| China, Japan & Korea | 4 | 0 |  | Ingman *et al.* ^20^ |
| Taiwan, China | 4 | 0 |  | Ingman *et al.*^21^ |
| Mongolia | 4 | 0 |  | Ingman *et al.*^22^ |
| Taiwan, China | 6 | 0 |  | Tabbada *et al.*^23^ |

Note - Population data were retrieved from the PhyloTree database ([http://www.phylotree.org](http://www.phylotree.org/); searched on April 29, 2011) but excluded problematic East Asian mtDNAs which were mentioned in Yao *et al.*^24^ LHON complete mtDNA sequences in the database were not considered.

^a^ GenBank accession numbers refer to mtDNA sequences bearing m.593T>C.

**Supplementary References**

1. Kong Q-P, Yao Y-G, Sun C, Bandelt H-J, Zhu CL, Zhang Y-P. Phylogeny of east Asian mitochondrial DNA lineages inferred from complete sequences. *Am J Hum Genet* 2003;**73**:671-6.

2. Kong Q-P, Bandelt H-J, Sun C, Yao Y-G, Salas A, Achilli A, Wang C-Y, Zhong L, Zhu CL, Wu SF, Torroni A, Zhang Y-P. Updating the East Asian mtDNA phylogeny: a prerequisite for the identification of pathogenic mutations. *Hum Mol Genet* 2006;**15**:2076-86.

3. Bandelt H-J, Achilli A, Kong Q-P, Salas A, Lutz-Bonengel S, Sun C, Zhang Y-P, Torroni A, Yao Y-G. Low "penetrance" of phylogenetic knowledge in mitochondrial disease studies. *Biochem Biophys Res Commun* 2005;**333**:122-30.

4. Wang C-Y, Wang H-W, Yao Y-G, Kong Q-P, Zhang Y-P. Somatic mutations of mitochondrial genome in early stage breast cancer. *Int J Cancer* 2007;**121**:1253-6.

5. Trejaut JA, Kivisild T, Loo JH, Lee CL, He CL, Hsu CJ, Lee ZY, Lin M. Traces of archaic mitochondrial lineages persist in Austronesian-speaking Formosan populations. *PLoS Biol* 2005;**3**:e247.

6. Tanaka M, Cabrera VM, González AM, et al. Mitochondrial genome variation in eastern Asia and the peopling of Japan. *Genome Res* 2004;**14**:1832-50.

7. Kong Q-P, Salas A, Sun C, Fuku N, Tanaka M, Zhong L, Wang C-Y, Yao Y-G, Bandelt H-J. Distilling artificial recombinants from large sets of complete mtDNA genomes. *PLoS One* 2008;**3**:e3016.

8. Kazuno A-A, Munakata K, Mori K, Tanaka M, Nanko S, Kunugi H, Umekage T, Tochigi M, Kohda K, Sasaki T, Akiyama T, Washizuka S, Kato N, Kato T. Mitochondrial DNA sequence analysis of patients with 'atypical psychosis'. *Psychiatry Clin Neurosci* 2005;**59**:497-503.

9. Bilal E, Rabadan R, Alexe G, Fuku N, Ueno H, Nishigaki Y, Fujita Y, Ito M, Arai Y, Hirose N, Ruckenstein A, Bhanot G, Tanaka M. Mitochondrial DNA haplogroup D4a is a marker for extreme longevity in Japan. *PLoS One* 2008;**3**:e2421.

10. Nohira C, Maruyama S, Minaguchi K. Phylogenetic classification of Japanese mtDNA assisted by complete mitochondrial DNA sequences. *Int J Legal Med* 2010;**124**:7-12.

11. Ueno H, Nishigaki Y, Kong Q-P, Fuku N, Kojima S, Iwata N, Ozaki N, Tanaka M. Analysis of mitochondrial DNA variants in Japanese patients with schizophrenia. *Mitochondrion* 2009;**9**:385-93.

12. Derenko M, Malyarchuk B, Grzybowski T, Denisova G, Dambueva I, Perkova M, Dorzhu C, Luzina F, Lee HK, Vanecek T, Villems R, Zakharov I. Phylogeographic analysis of mitochondrial DNA in northern Asian populations. *Am J Hum Genet* 2007;**81**:1025-41.

13. Zhao M, Kong Q-P, Wang H-W, Peng M-S, Xie X-D, Wang W-Z, Jiayang, Duan J-G, Cai M-C, Zhao S-N, Cidanpingcuo, Tu Y-Q, Wu S-F, Yao Y-G, Bandelt H-J, Zhang Y-P. Mitochondrial genome evidence reveals successful Late Paleolithic settlement on the Tibetan Plateau. *Proc Natl Acad Sci U S A* 2009;**106**:21230-5.

14. Peng M-S, Quang H-H, Dang K-P, Trieu A-V, Wang H-W, Yao Y-G, Kong Q-P, Zhang Y-P. Tracing the Austronesian footprint in Mainland Southeast Asia: a perspective from mitochondrial DNA. *Mol Biol Evol* 2010;**27**:2417-30.

15. Bi R, Zhang A-M, Zhang W, Kong Q-P, Wu BL, Yang X-H, Wang D, Zou Y, Zhang Y-P, Yao Y-G. The acquisition of an inheritable 50-bp deletion in the human mtDNA control region does not affect the mtDNA copy number in peripheral blood cells. *Hum Mutat* 2010;**31**:538-43.

16. Kong Q-P, Sun C, Wang H-W, Zhao M, Wang W-Z, Zhong L, Hao X-D, Pan H, Wang S-Y, Cheng Y-T, Zhu C-L, Wu S-F, Liu L-N, Jin J-Q, Yao Y-G, Zhang Y-P. Large-scale mtDNA screening reveals a surprising matrilineal complexity in east Asia and its implications to the peopling of the region. *Mol Biol Evol* 2011;**28**:513-22.

17. Peng M-S, Palanichamy M-G, Yao Y-G, Mitra B, Cheng Y-T, Zhao M, Liu J, Wang H-W, Pan H, Wang W-Z, Zhang A-M, Zhang W, Wang D, Zou Y, Yang Y, Chaudhuri T-K, Kong Q-P, Zhang Y-P. Inland post-glacial dispersal in East Asia revealed by mitochondrial haplogroup M9a'b. *BMC Biol* 2011;**9**:2.

18. Qin Z, Yang Y, Kang L, Yan S, Cho K, Cai X, Lu Y, Zheng H, Zhu D, Fei D, Li S, Jin L, Li H. A mitochondrial revelation of early human migrations to the Tibetan Plateau before and after the last glacial maximum. *Am J Phys Anthropol* 2010;**143**:555-69.

19. Hartmann A, Thieme M, Nanduri LK, Stempfl T, Moehle C, Kivisild T, Oefner PJ. Validation of microarray-based resequencing of 93 worldwide mitochondrial genomes. *Hum Mutat* 2009;**30**:115-22.

20. Ingman M, Kaessmann H, Paabo S, Gyllensten U. Mitochondrial genome variation and the origin of modern humans. *Nature* 2000;**408**:708-13.

21. Ingman M, Gyllensten U. Mitochondrial genome variation and evolutionary history of Australian and New Guinean aborigines. *Genome Res* 2003;**13**:1600-6.

22. Ingman M, Gyllensten U. Rate variation between mitochondrial domains and adaptive evolution in humans. *Hum Mol Genet* 2007;**16**:2281-7.

23. Tabbada KA, Trejaut J, Loo J-H, Chen Y-M, Lin M, Mirazon-Lahr M, Kivisild T, De Ungria MC. Philippine mitochondrial DNA diversity: a populated viaduct between Taiwan and Indonesia? *Mol Biol Evol* 2010;**27**:21-31.

24. Yao Y-G, Salas A, Logan I, Bandelt H-J. mtDNA data mining in GenBank needs surveying. *Am J Hum Genet* 2009;**85**:929-33.
