# Supplementary material for: Is Mitochondrial tRNAphe Variant m.593T>C a Synergistically Pathogenic Mutation in Chinese LHON Families with m.11778G>A?
Source: PLoS One. 2011 Oct 19;6(10):e26511. doi: 10.1371/journal.pone.0026511 (PMC3198432; doi:10.1371/journal.pone.0026511)
Supplement: Table S2 — mtDNA sequence variation of 75 individuals with m.593T>C. (PDF) [file pone.0026511.s002.pdf]

Table S2 mtDNA sequence variation of 75 individuals with m.593T>C

Group	Sample	Haplogroup	Segment I (16000+)	Segment II	5176*Alu* I	region 1 (4887-5442)	region 2 (10170-10660)	Reference
Han Chinese from Hunan Province	HN-SZ364	B5a1c	140 182C 183C 189 266A 519	73 210 263 309+2C 315+C 501 523-524d 593 709 750		5178C, 5237A	10325A, 10398G, 10523G	this study
	HN-CT376	B5a1c	140 183C 189 240 266A 519	73 210 263 315+C 523-524d 593 709 750 827		5178C, 5237A	10325A, 10398G, 10523G	this study
	HN-SZ366	B5a1c	140 183C 189 262 266A 274 519	64 73 210 263 315+C 523-524d 593 709 750		5178C, 5237A	10325A, 10398G, 10523G	this study
	HN-SZ339	B5a1c	140 183C 189 262 266A 519	64 73 210 263 309+C 315+C 523-524d 593 709 750		5178C, 5237A	10325A, 10398G, 10523G	this study
	HN-SZ21	B5a1c	140 183C 189 262 266A 519	73 210 263 309+C 315+C 523-524d 593 709 750		5178C, 5237A	10325A, 10398G, 10523G	this study
	HN-SZ376	B5a1c	140 183C 189 266A 294 519	73 210 263 309+2C 315+C 523-524d 593 709 750		5178C, 5237A	10325A, 10398G, 10523G	this study
	HN-SZ730	B5a	140 183C 189 266A 311 519	73 152 210 263 309+2C 315+C 471 523-524d 593 709 750		5178C, 5237G	10325G, 10398G, 10523A	this study
	HN-CT350	B5a1c	140 183C 189 266A 519	73 210 263 309+2C 315+C 523-524d 593 709 750		5178C, 5237A	10325A, 10398G, 10523G	this study
	HN-SZ12	B5a1c	140 183C 189 266A 519	73 210 263 309+C 315+C 523-524d 593 709 750		5178C, 5237A	10325A, 10398G, 10523G	this study
	HN-SZ136	B5a1c	140 183C 189 266A 519	73 210 263 309+C 315+C 523-524d 593 709 750		5178C, 5237A	10325A, 10398G, 10523G	this study
	HN-SZ239	B5a1c	140 183C 189 266A 519	73 210 263 309+C 315+C 523-524d 593 709 750		5178C, 5237A	10325A, 10398G, 10523G	this study
	HN-SZ246	B5a1c	140 183C 189 266A 519	73 210 263 309+C 315+C 523-524d 593 709 750		5178C, 5237A	10325A, 10398G, 10523G	this study
	HN-SZ558	B5a1c	140 183C 189 266A 519	73 210 263 309+C 315+C 523-524d 593 709 750		5178C, 5237A	10325A, 10398G, 10523G	this study
	HN-SZ637	B5a1c	140 183C 189 266A 519	73 210 263 309+C 315+C 523-524d 593 709 750		5178C, 5237A	10325A, 10398G, 10523G	this study
	HN-CT4	B5a1c	140 183C 189 266A 519	73 210 263 315+C 523-524d 593 709 750		5178C, 5237A	10325A, 10398G, 10523G	this study
	HN-CT85	B5a1c	93 140 183C 189 262 266A 519	64 73 210 263 315+C 523-524d 593 709 750		5178C, 5237A	10325A, 10398G, 10523G	this study
	HN-CT207	C	223 298 327 519	73 249d 263 315+C 489 593 750				this study
	HN-CT235	F1a1	129 162 172 261 304 519	73 195 249d 263 315+C 523-524d 593 750				this study
	HN-CT569	G1c	220 223 362 519	73 263 309+C 315+C 372 489 593 709 750	+			this study
	HN-CT317	G1c	223 261 362 519	73 200 263 315+C 489 593 709 750	+			this study
	HN-CT639	G1c	223 261 362 519	73 263 315+C 489 523-524d 593 709 750	+			this study
	HN-SZ230	G1c	223 319 362 519	73 263 315+C 408A 489 593 709 750				this study
	HN-CT691	G1c	223 362 399 519	73 263 309+C 315+C 374 489 593 709 750	+			this study
	HN-CT220	G1c	223 362 519	73 152 263 315+C 489 593 709 750	+			this study
	HN-CT229	G1c	223 362 519	73 263 315+C 489 593 709 750				this study
	HN-CT73	G1c	51 189 223 253 362 519	73 263 309+C 315+C 489 593 709 750	+			this study
	HN-CT629	G1c	51 189 223 362 519	73 185 188 263 309+C 315+C 489 593 709 750	+			this study
	HN-SZ33	G1c	51 189 223 362 519	73 263 309+C 315+C 489 593 709 750	+			this study
	HN-SZ192	M7b1	129 189 192 223 248 297 519	73 150 199 263 309+C 315+C 489 593 750				this study
	HN-CT270	M7c	295 319 519	73 146 199 263 315+C 489 523-524d 593 750				this study
Han Chinese from Yunnan Province	LP-YM11	A	92 223 290 311 319	64 73 146 235 263 309+C 315+C 522-523d 593 663 750				this study
	LP-TH20	B5a1c1	140 182C 183C 189 242A 266A 519	73 210 263 315+C 523-524d 593 709 750		5178C, 5237A	10325A, 10398G, 10523G	this study
	LP-HN96	B5a1c1	140 182C 183C 189 266A 519	73 210 263 315+C 522-523d 593 709 750		5178C, 5237A	10325A, 10398G, 10523G	this study
	HTC21	B5a1c1	140 183C 189 262 266A 519	64 73 210 263 309+C 315+C 393d 523-524d 593 709 750		5178C, 5237A	10325A, 10398G, 10523G	this study
	LP-JC33	B5a1c1	140 183C 189 262 266A 519	64 73 210 263 315+C 523-524d 593 709 750		5178C, 5237A	10325A, 10398G, 10523G	this study
	HNC195	B5a1c1	140 183C 189 262 266A 519	64 73 210 263 315+C 522-523d 593 709 750		5178C, 5237A	10325A, 10398G, 10523G	this study
	YJC05	B5a1c1	140 183C 189 262 266A 519	64 73 81 210 263 309+2C 315+C 522-523d 593 709 750		5178C, 5237A	10325A, 10398G, 10523G	this study
	HNC81	B5a1c1	140 183C 189 266A 519	73 210 263 309+C 315+C 522-523d 593 709 750		5178C, 5237A	10325A, 10398G, 10523G	this study
	JCC70	B5a1c1	140 183C 189 266A 519	73 210 263 315+C 522-523d 593 709 750		5178C, 5237A	10325A, 10398G, 10523G	this study
	HNC196	F1a1	129 162 172 304 519	73 195 249d 263 315+C 522-523d 593 750				this study
	LP-ES17	G1c	103h 223 362 519	73 263 315+C 489 593 709 750	+			this study
	JCC117	G1c	126 223 362 519	73 263 315+C 489 593 709 750	+			this study
	LP-YM27	G1c	223 362 474C 519	73 263 309+2C 315+C 455+T 489 593 709 750	+			this study
	LP-JC55	G1c	223 362 519	73 263 309+C 315+C 489 593 709 750	+			this study
	THC36	G1c	223 362 519	73 153 263 309+CC 315+C 489 593 709 750	+			this study
	LP-TH85	G1c	223 362 519	73 263 309+C 315+C 489 593 709 750	+			this study

	LP-YM25	G1c	223 362 519	73 263 315+C 489 593 709 750	+			this study
	YMC89	G1c	93 223 362 519	73 263 309+C 315+C 489 593 709 723 750	+			this study
	XPC11	M7b1	129 189 192 223 297 519	73 150 199 263 309+C 315+C 489 593 750				this study
Suspected LHON patient	Le728	B5a1c1	140 182C 183C 189 266A 519	73 210 263 315+C 523-524dAC 593 709 750		5178C, 5237A	10325A, 10398G, 10523G	this study
	Le1201	B5a1c1	140 183C 189 262 266A 519	64 73 210 263 315+C 523-524dAC 593 709 750		5178C, 5237A	10325A, 10398G, 10523G	this study
	Le850	B5a1c1	140 183C 189 262 266A 519	73 210 263 315+C 523-524dAC 593 709 750		5178C, 5237A	10325A, 10398G, 10523G	this study
	Le690	B5a1c1	140 183C 189 266A 519	73 210 263 309+C 315+C 523-524dAC 593 709 750		5178C, 5237A	10325A, 10398G, 10523G	this study
	Le1204	B5a1c1	140 183C 189 266A 519	73 210 263 315+C 523-524dAC 593 709 750		5178C, 5237A	10325A, 10398G, 10523G	this study
	Le1465	D4g2	223 271 362 519	73 263 298 315+C 489 593 750	-			this study
	Le15(1)	G1c	223 362 519	73 263 309+C 315+C 489 593 709 750	+			this study
	Le433	G1c	92 223 362 519	73 263 315+C 489 593 709 750	+			this study
	Le709	M7b1	129 189 192 223 278 297 519	73 150 199 263 315+C 489 593 750				this study
	Le949	M7b1	129 189 192 223 297 519	73 150 199 263 309+C 315+C 489 593 750				this study
	Le636	M7b1	144 189 192 223 297 519	73 150 199 263 315+C 489 593 750				this study
	Le933	M7b1'2	129 189 223 297 319 519	73 150 199 263 309+C 315+C 489 593 750				this study
LHON patient	Le1407	B5a1c1	140 183C 189 262 266A 519	73 210 263 309+C 315+C 523-524d 593 709 750		5178C, 5237A	10325A, 10398G, 10523G	this study
	Le1192	B5a1c1	140 183C 189 262 266A 519	64 73 210 263 309+C 315+C 523-524dAC 593 709 750		5178C, 5237A	10325A, 10398G, 10523G	this study
	Le51	C7a2	183C 189 223 298 327 519	73 249d 263 309+CC 315+C 489 593 750				this study
	Le394	D4	192 223 362 519	73 263 309+C 315+C 489 593 709 750	-			Ji et al. 2008
	Le1120	D4g2	223 271 362 519	73 263 298 315+C 489 593 750	-			Ji et al. 2008
	Le840	D4g2	223 271 362 519	73 263 298 315+C 489 593 750	-			this study
	Le878	G1c	189 223 319 362 519	73 263 309+C 315+C 489 593 709 750	+			this study
	Le1561	G1c	223 284 295 297 362 519	73 146 207 263 309+CC 315+C 489 593 709 750	+			this study
	Le953	G1c	223 362 519	73 263 309+C 315+C 489 593 709 750	+			Ji et al. 2008
	Le682	G1c	223 362 519	73 263 315+C 489 593 709 750	+			this study
	Le554	G1c	93 223 362 519	73 263 315+C 489 593 709 750	+			Ji et al. 2008
	Le549	M10a2	66 220 223 311 519	73 263 315+C 489 573+4C 593 709 750				Ji et al. 2008
	Le53	M7b1'2'4	129 189 223 297 519	73 150 199 263 315+C 489 593 750				this study
	Le251	Z	185 223 260 298 311 519	73 152 249d 263 309+CC 315+C 489 593 709 750				Ji et al. 2008

Note – Sequence variation was scored relative to the revised Cambridge reference sequence (rCRS, Andrews et al. 1999). Suffixes A, G, C and T indicate transversions, "h" indicates heteroplasmy, "d" and "+" indicate deletions and insertions, respectively. Indels (insertion and deletion) are recorded at the last possible site. "+" and "－" denote the absence and presence of the restriction site, respectively. Sample Le251 was wrongly classified as M8a1 in Ji et al. (2008) and the correct classification should be Z. Note that another sample in Ji et al. (2008), Le1357, was wrongly written as D5b2b, but it should be D4b2b or better to be treated as D4 based on the available sequence variation.

Supplementary References

Andrews RM, Kubacka I, Chinnery PF, Lightowlers RN, Turnbull DM, Howell N. Reanalysis and revision of the Cambridge reference sequence for human mitochondrial DNA. Nat Genet 1999;23:147

Ji Y, Zhang A-M, Jia X, Zhang Y-P, Xiao X, Li S, Guo X, Bandelt H-J, Zhang Q, Yao Y-G. Mitochondrial DNA haplogroups M7b1'2 and M8a affect clinical expression of leber hereditary optic neuropathy in Chinese families with the m.11778G>A mutation. Am J Hum Genet 2008; 83: 760-768
